# Tumor flare reaction in a patient with mycosis fungoides treated with a novel immune-epigenetic doublet

**DOI:** 10.1016/j.jdcr.2024.03.016

**Published:** 2024-04-07

**Authors:** Nakul Dar, Nathan Roberts, Alejandro Gru, Ifeyinwa Obiorah, Jiefu Zheng, Lale Kostakoglu Shields, Thomas Cropley, Enrica Marchi

**Affiliations:** a University of Virginia School of Medicine, Charlottesville, Virginia; b Department of Hematology and Oncology, University of Virginia Comprehensive Cancer Center, Charlottesville, Virginia; c Department of Dermatology, Columbia University Irvine Medical Center, New York, New York; d Department of Pathology, University of Virginia Medical Center, Charlottesville, Virginia; e Department of Radiology and Medical Imaging, University of Virginia Medical Center, Charlottesville, Virginia; f Department of Dermatology, University of Virginia Medical Center, Charlottesville, Virginia

**Keywords:** aggressive non-Hodgkin's lymphoma, cell therapy and immunotherapy, cutaneous T-cell lymphoma, flare reaction, lymphoproliferative disorders, mycosis fungoides, pembrolizumab

## Introduction

Folliculotropic mycosis fungoides (FMF) is a rare cutaneous T-cell lymphoma that demonstrates preferential infiltration of the follicular epithelium and hair follicles. Tumor flare reactions (TFRs) have been described in solid tumors and other nodal lymphomas treated with anti-programmed cell death protein-1 (PD-1) therapies, but less is known regarding the incidence and significance of TFR in patients with cutaneous T-cell lymphoma. We report a case of TFR in a patient with FMF who was treated with decitabine and pembrolizumab.

## Case presentation

**Fig 1:**
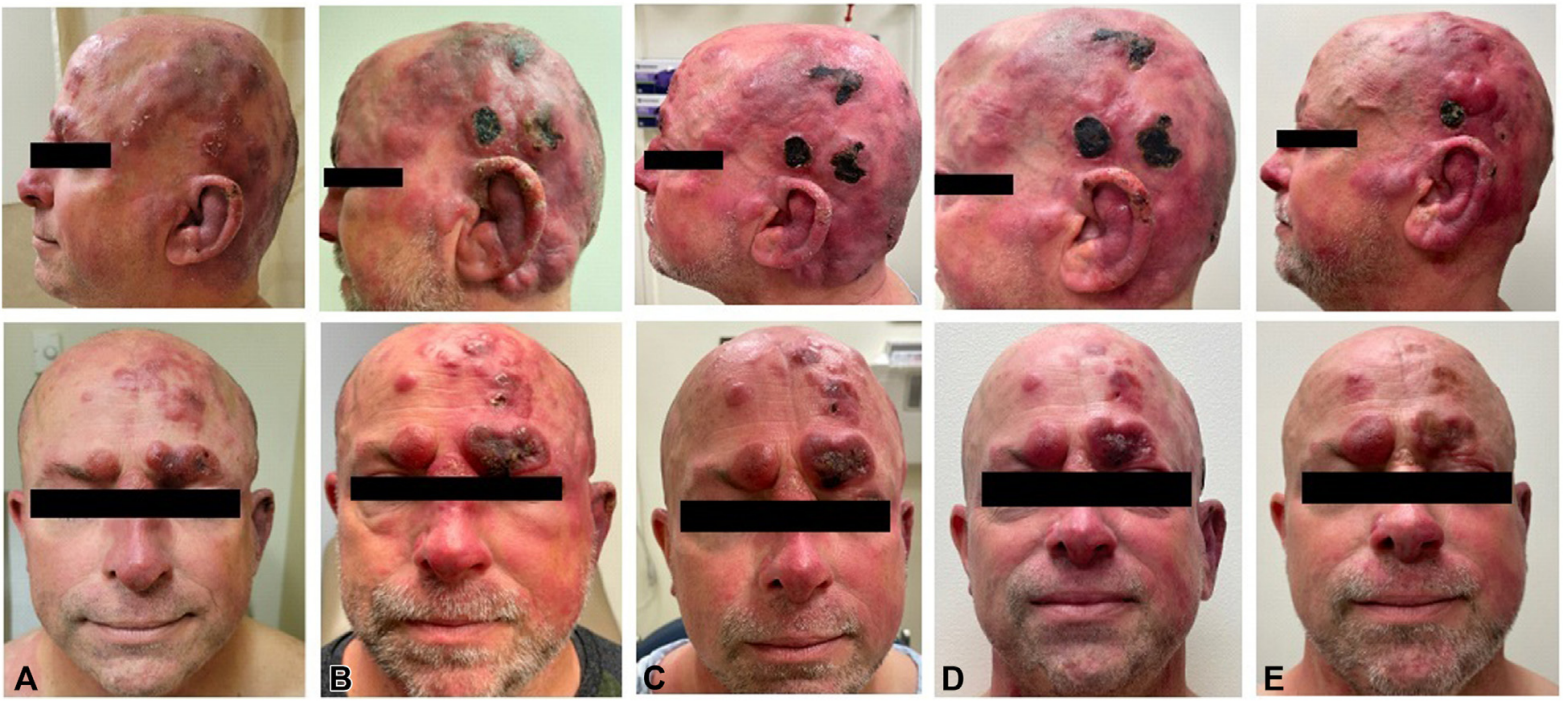
Progression of scalp disease from pretreatment (**A**) to days 6 (**B**), 13 (**C**), 20 (**D**), and 76 (**E**) following first pembrolizumab administration.

**Fig 2:**
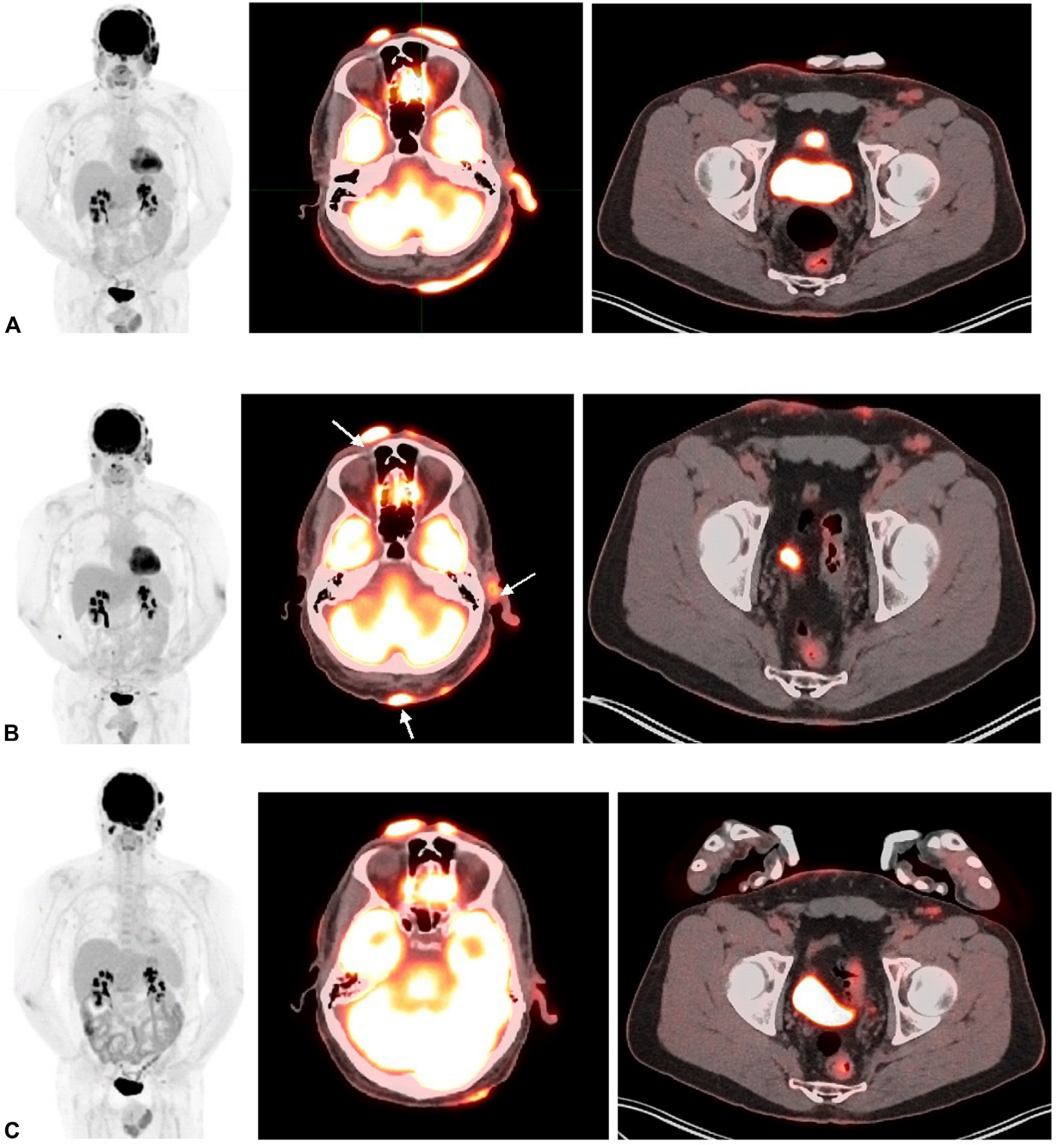
^18^F-FDG PET/CT results pretreatment (**A**) and following 2 (**B**) and 4 cycles of treatment (**C**). **A,** Numerous hypermetabolic cutaneous lesions throughout the scalp, most prominent on the *left* with involvement of the *left* ear (SUV_max_ up to 16 in the occipital region, *white arrows*, *middle* panel). Mild cutaneous thickening with mild FDG uptake in lower anterior abdominal wall is noted (*white arrow*, *right* panel). Mildly hypermetabolic lymphadenopathy in the bilateral neck, axillary, and inguinal regions. **B,** Significant interval decreases in size and FDG uptake of the scalp lesions, with some lesions completely resolved. Some lesions show interval worsening (ie, *right* nasal ala lesion, SUV_max_ 8.2, previously 3; *left* preauricular lesion, SUV_max_ 8, previously 3.3, *white arrows*, *middle* panel). There is slight interval increase in size of hypermetabolic skin thickening and new subcutaneous soft tissue nodules in the lower abdominal region. There is grossly stable mildly hypermetabolic lymphadenopathy in the bilateral neck, axillary, and inguinal regions. **C,** Mixed interval changes regarding the multifocal cutaneous scalp hypermetabolic lesions as some are improved while others are worsened. Improved anterior lower abdominal wall cutaneous hypermetabolic lesions. Grossly stable mildly hypermetabolic bilateral cervical lymph nodes. Slightly less conspicuous FDG uptake in bilateral axillary and inguinal lymph nodes. *^18^F-FDG PET/CT*, 18F-fluorodeoxyglucose positron emission tomography-computed tomography; *SUV*_*max*_, maximum standardized uptake value.

**Fig 3:**
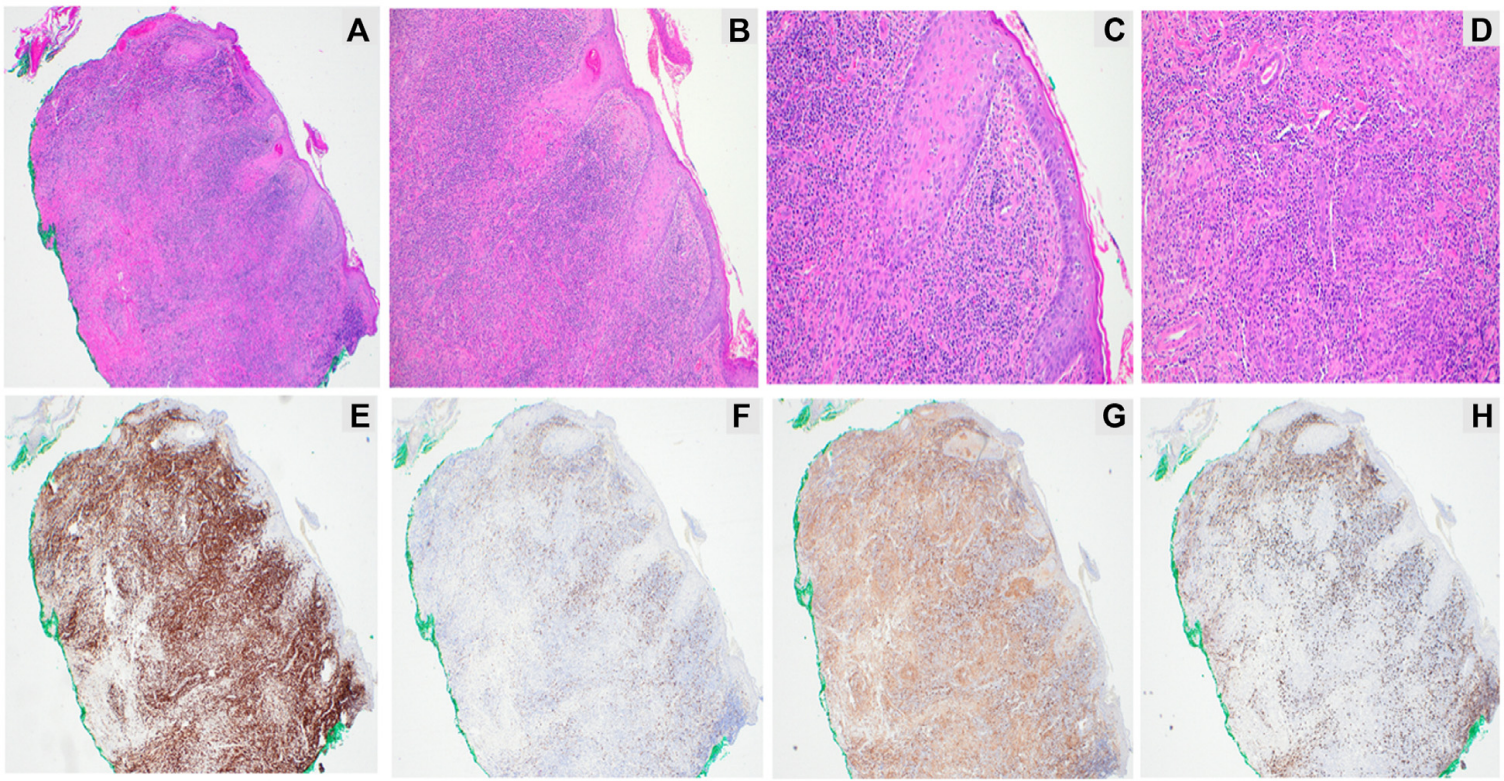
Skin biopsy collected prior to treatment with pembrolizumab and decitabine. Hematoxylin and eosin-stained slides (**A**-**D**) show a dense dermal lymphocytic infiltrate with limited epidermotropic and significant folliculotropism. Immunochemical stains demonstrate positivity with CD3 (**E**) and markedly decreased expression of CD7 (**F**), CD4 (**G**), and CD8 (**H**).

**Fig 4:**
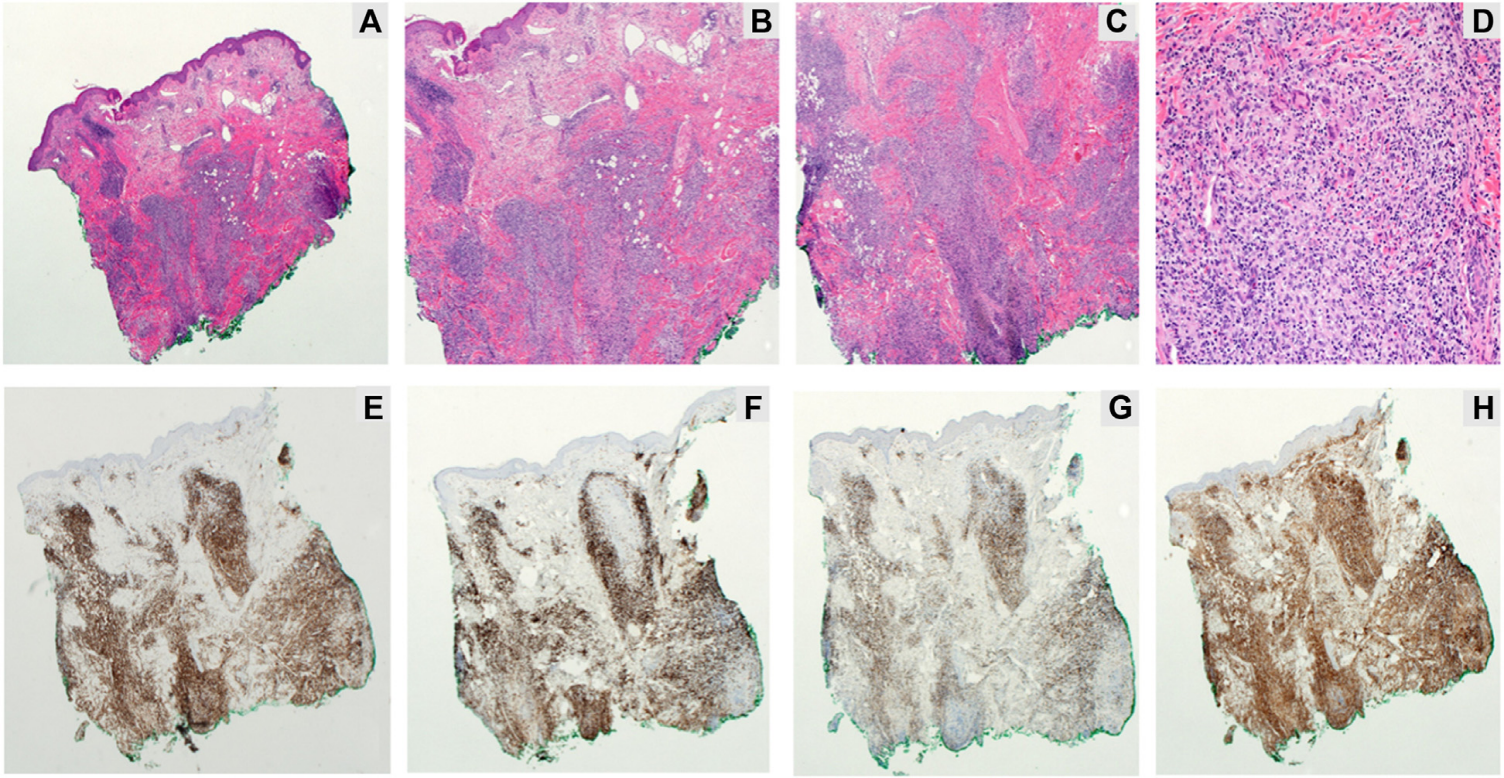
Skin biopsy collected 6 days after first pembrolizumab administration during tumor flare. Hematoxylin and eosin-stained slides (**A**-**D**) demonstrate dermal perifollicular lymphocytic infiltrates and folliculotropism, composed of small atypical lymphoid cells, eosinophils, and granulomatous inflammation. On immunohistochemistry, the lymphoid cells are predominantly positive for CD3 (**E**) and CD8 (**F**) with mild decreased expression of CD7 (**G**). CD4 staining (**H**) highlights the increased histiocytic proliferation.

A 52-year-old male with a 14-year history of FMF presented following disease progression after multiple therapies. He was previously treated with skin-directed therapies including psoralen plus ultraviolet A and local electron beam therapy, as well as systemic therapies including gemcitabine, pralatrexate, and romidepsin. The patient agreed to enroll in a phase 1B trial, PTCL-002 (ClinicalTrials.gov identifier: NCT03240211). He exhibited tumor-stage lesions involving the left ear, eyebrow, scalp, and nose (Fig 1, *A*). Positron emission tomography–computed tomography demonstrated hypermetabolic cutaneous lesions throughout the scalp (Fig 2, *A*). A punch biopsy of a tumor-stage lesion above the left eyebrow revealed a superficial and deep dermal lymphoid infiltrate with focal follicular and perifollicular distribution (Fig 3, *A*-*D*). Immunohistochemical staining (Fig 3, *E*-*H*) showed that the lymphoid cells, highlighted by cluster of differentiation (CD)3, had decreased or absent expression of CD4, CD7, and CD8. These results were consistent with FMF with no evidence of large cell transformation. He was started on decitabine 20 mg/m^2^ on days 1 to 3 administered every 28 days and pembrolizumab 200 mg on day 8 administered every 21 days. He tolerated initial decitabine infusions well with no adverse events. However, within 24 hours of first pembrolizumab infusion, he developed severe pain with worsening erythema and edema at all sites of cutaneous disease with worst symptoms affecting the scalp, left ear, and eyebrows (Fig 1, *B*). A punch biopsy of a scalp lesion was obtained 6 days after first pembrolizumab infusion. This showed a superficial and deep dermal lymphoid infiltrate with perifollicular accentuation and prominent infiltration of the hair follicle epithelium (Fig 4). The infiltrate was comprised of CD8+ atypical lymphoid cells admixed with new findings of increased eosinophils and areas of granulomatous inflammation. Due to concern for TFR, he was treated with oral prednisone 10 mg daily with rapid symptom relief. At follow up 1 week later, multiple tumor lesions appeared to have flattened and decreased in size with development of thick necrotic eschar (Fig 1, *C*). He completed a 10-day course of corticosteroids. Treatment was resumed without interruption or dose modification, and no additional flares or adverse events occurred. He demonstrated improvement in cutaneous disease severity as assessed by modified severity-weighted assessment score, which improved from 41.8 to 24.2 by cycle 7. Positron emission tomography–computed tomography after cycle 2 demonstrated stable disease with findings of reduced size and avidity of multiple scalp lesions (Fig 2, *B*). Trial participation was discontinued after cycle 7 due to overt disease progression. He was subsequently started on brentuximab vedotin as bridging therapy prior to allogeneic stem cell transplantation.

## Discussion

Mycosis fungoides (MF) is the most common primary cutaneous T-cell lymphoma and originates from peripheral epidermotropic memory T-cells that are typically CD4+.^1^ Several distinct clinicopathologic variants are recognized.^2^ In the case of FMF, histopathology reveals an abnormal lymphocytic infiltrate with a variable degree of folliculotropism and sometimes follicular mucinosis. Early-stage disease is treated with skin-directed therapies, including topical corticosteroids, chemotherapeutic agents, retinoids, or phototherapy.^3^ Treatment of advanced-stage disease involves systemic chemotherapy, targeted agents, and/or radiation and typically requires a multidisciplinary approach with input from dermatologists and medical oncologists.^4^

Biomarkers such as CD30 are important for diagnostic and therapeutic decision making. In addition, PD-1 and programmed cell death ligand 1, represent potential therapeutic targets. PD-1 expression on malignant T cells in MF and Sézary syndrome is highly variable.^5^ The therapeutic potential of the anti-PD-1 monoclonal antibodies, nivolumab, and pembrolizumab, has been described in early phase clinical trials.^6^^,^^7^ In a phase 1 study of relapsed/refractory MF, treatment with nivolumab produced an overall response rate of 15%.^6^ In a similar phase 2 study, treatment with pembrolizumab produced an overall response rate of 38%. In the pembrolizumab study, a transient worsening of erythroderma and pruritus, termed “cutaneous flare reaction,” was observed in 53% of patients with Sézary syndrome.^7^ This flare reaction is hypothesized to occur as a result of increased activation of CD8+ effector T cells mediated by PD-1 inhibition as opposed to true disease progression.^8^ In patients with MF, TFR can be challenging to differentiate from progression of disease on clinical assessment alone. There is no current evidence to suggest that TFR is more likely to occur in FMF versus other MF subtypes. Flare reactions have similarly been observed in patients with MF treated with other immunomodulatory agents. For example, in a phase 2 trial evaluating lenalidomide in relapsed/refractory MF or Sézary syndrome, flare reactions were reported in 25% of patients, with flare potentially correlating with favorable treatment response.^9^

The constellation of clinical and pathological findings in our patient supports the hypothesis of TFR. The presence of a CD8+ predominant infiltrate without aberrant loss of other T-cell markers was consistent with increased cytotoxic activity. In contrast, skin biopsy in cases of disease progression typically shows increased infiltration by CD4+ T cells with aberrant loss of other maturation markers such as CD7. Whether or not decitabine increased the likelihood of TFR remains unclear, and future studies are needed to evaluate the synergistic effects of hypomethylating agents on anti-PD-1 therapies. Following the flare reaction, our patient experienced clinical benefit with reduction in symptoms and improvement in modified severity-weighted assessment score although the absolute improvement in modified severity-weighted assessment did not meet strict criteria for partial response.^10^ His eventual disease progression raises questions about the predictive value of TFR. Oncologists and dermatologists monitoring patients with MF receiving anti-PD-1 therapies should be aware of the potential for TFR to occur. In such cases, prompt biopsy with review by a dermatopathologist with expertise in cutaneous lymphoid malignancies can aid in distinguishing flare reaction from disease progression. As shown in this case, patients with TFR may be safely managed with low-dose oral corticosteroids without major disruptions to their disease management.

## Conflicts of interest

Dr Gru reports participation on a Data Safety Monitoring Board or Advisory Board of StemLine, Seattle Genetics, and Innate Pharma; receives payment or honoraria for lectures, presentations, speakers bureaus, manuscript writing, or educational events from Kyowa Kyrin and StemLine Therapeutic. Dr Marchi is a scientific advisor for Kyowa Kirin, Seagen, and Acrotech. Author Dar and Drs Roberts, Obiorah, Zheng, Shields, and Cropley have no conflicts of interest to declare.
